# 

**Published:** 1958-01

**Authors:** 


### THE MEDICAL JOURNAL OF THE SOUTH-WEST

*Incorporating*
*The Bristol Medico-Chirurgical Journal*

PUBLISHED UNDER THE AUSPICES OF THE
BRISTOL MEDICO-CHIRURGICAL SOCIETY

*1958 Vol. 73*

J. W. ARROWSMITH LTD., WINTERSTOKE ROAD, BRISTOL

### *1958 Contents of Vol. 73*

| | *page* |
|---|---|
| Foreword. By Sir PHILIP MORRIS, C.B.E., M.A., LL.D. | 1 |
| Some Thoughts of Medical Education. By C. BRUCE PERRY, M.D., F.R.C.P. | 3 |
| The Medical Library of the University of Bristol. By A. E. S. ROBERTS | 12 |
| Carcinoma of the Thyroid. (A Review of 100 Cases). By J. W. BRADBEER, M.B., F.R.C.S. | 15 |
| Kyphoscoliosis with Pulmonary Hypertension (Clinical Pathological Conference) | 22 |
| Diseases in Search of Viruses. By C. H. STUART-HARRIS, M.D., F.R.C.P. | 29 |
| Pain Related to the Teeth and Jaws. By A. I. DARLING | 36 |
| Supportive Treatment of Acute Cor Pulmonale due to Massive Pulmonary Embolism. By E. A. W. HOUGHTON, M.B., CH.B. | 41 |
| Accidental Poisoning with the Antihistamine Drug "Histantin" (Clinical Pathological Conference) | 45 |
| Hospital Cross-Infection. By W. A. GILLESPIE, M.D., F.R.C.P.I., D.P.H. | 56 |
| On Growing Old. By J. H. SHELDON, C.B.E., M.D., F.R.C.P. | 69 |
| Meningococcal Septicaemia with Bilateral Adrenal Haemorrhage (Waterhouse-Friderichsen Syndrome) | 78 |
| The Investigation of Persistent Diarrhoea. By JOHN NAISH, M.D., F.R.C.P. | 87 |
| Some Modern Developments in Peripheral Vascular Surgery. By R. E. HORTON, M.B.E., M.S., F.R.C.S. | 92 |
| Perinatal Mortality. By THOMAS E. OPPÉ, M.B., M.R.C.P. | 99 |
| An Exhibition of Important Anatomical Books. By A. E. S. ROBERTS | 104 |
| Hereditary Telangiectasia: Hodgkin's Disease | 108 |
| CORRESPONDENCE | 26, 50 |
| NOTES AND NEWS | 26, 51, 82, 116 |
| APPOINTMENTS | 27, 53, 83, 117 |
| NEWS FROM SOCIETIES | 115 |

THE

MEDICAL JOURNAL OF THE SOUTH-WEST

*(Incorporating the Bristol Medico-Chirurgical Journal)*

Established 1883

VOL. 73 (i) January 1958 NO. 267

### Contents

| | | Page |
|---|---|---|
| FOREWORD | *Sir Philip Morris* | 1 |
| SOME THOUGHTS OF MEDICAL EDUCATION | *Prof. C. Bruce Perry* | 3 |
| THE MEDICAL LIBRARY OF THE UNIVERSITY OF BRISTOL | *Mr. A. E. S. Roberts* | 12 |
| CARCINOMA OF THE THYROID | *Mr. J. W. Bradbeer* | 15 |
| KYPHOSCOLIOSIS WITH PULMONARY HYPERTENSION | *Clinical Pathological Conference* | 22 |
| CORRESPONDENCE | | 26 |
| NOTES AND NEWS | | 26 |
| APPOINTMENTS | | 27 |

*PUBLISHED QUARTERLY*

*ANNUAL SUBSCRIPTION 16/- POST FREE*

PUBLISHED UNDER THE AUSPICES OF THE
BRISTOL MEDICO-CHIRURGICAL SOCIETY

### Editorial Committee

Dr. J. E. Cates, Department of Medicine, Bristol Royal Infirmary. *Editor.*
Dr. N. J. Brown, Southmead Hospital, Bristol. *Assistant Editor.*
Dr. J. Macrae, Ham Green Hospital.
Dr. R. C. Wofinden, Central Clinic, Bristol.
Mr. A. E. S. Roberts, Medical Library, University of Bristol. *Secretary.*

### *Local Correspondents*

*Bath* . . Mr. A. D. Bateman, 3 The Circus, Bath.
*Exeter* . . Dr. L. N. Jackson, Mount Jocelyn, Crediton, Devon.
*Gloucester* . Dr. R. F. Jarrett, 9 College Green, Gloucester.
*Plymouth* . Dr. C. R. Croft, Lexden, Hartley Av., Plymouth.
*Taunton* . Dr. M. McAlley, 1 The Crescent, Taunton.
*Torquay* . Dr. A. Robinson Thomas, 20 Devon Square, Newton Abbot, Devon.
*Truro* . . Dr. F. D. M. Hocking, St. Andrews, Perranporth, Cornwall.
*Yeovil* . . Mr. J. A. Vere Nicoll, Knapp House, Yeovil, Somerset.

### NOTICE TO CONTRIBUTORS

Original articles are invited, provided they have not been published elsewhere. Notes of forthcoming events, meetings held, degrees conferred, staff appointments, and other local news are welcomed. The editorial committee reserves the right to make literary corrections.

Illustrations should always be supplied ready for reproduction. All re-touchings or other operations required will be charged to the author. Line drawings should be drawn in Indian ink. We regret that we must ask authors to pay for making blocks, if this is necessary.

J. W. ARROWSMITH LTD., WINTERSTOKE ROAD,
BRISTOL.

### *Correspondence*

### *Notes and News*

Mr. W. M. Capper, M.B. (Lond.), F.R.C.S., M.R.C.O.G., has been elected a member of the court of examiners of the Royal College of Surgeons of England.

Mr. Keith Chitty, B.A., M.B. (Camb.), F.R.C.S., has been appointed consultant surgeon, Mid-Worcestershire group of hospitals.

Mr. P. C. Watson, M.B. (Lond.), F.R.C.S. has been appointed consultant general surgeon, Boston area hospitals.

### *EXAMINATION RESULTS*

*L.R.C.P., M.R.C.S.:* V. P. Nagpal.

### *UNIVERSITY OF BRISTOL*

M.B., Ch.B.

*DECEMBER* 1957

PASS

Addy, M. G.

Bruton, C. N.

Codd, A. A.

Cole, J. C. G.

Edmondson, J. S.

Gibbins, R. R.

Howton, D. H.

Lartey, O.

Meadows, J. G.

Roper, K. J.

Russell, G. A.

Weeks, R. F.

Williams, R. C.

### *OBITUARY*

Dr. J. M. Cormack, Dr. W. A. L. Holland, Dr. George Struthers, Mr. R. Warren.

**Receipts for Subscriptions.**

In future when subscriptions to the Society are paid by cheque the Hon. Treasurer will send no receipt for subscriptions, unless a receipt is specifically asked for.

The step is covered by the Cheque Act, 1957, Section 3, which provides that even an un-endorsed cheque which appears to have been paid is evidence of receipt.

In this way, the Society will be saved over £7 0s. 0d. a year in postage alone.

### *Appointments*

### *UNITED BRISTOL HOSPITALS*

*October to November*, 1957

| *Name* | *Appointment* | *Formerly* |
|---|---|---|
| M. W. Reece, F.R.C.S. | Senior Surgical Registrar. | Surgical Registrar, Exeter. |
| W. Wypkema, F.R.C.S. | Registrar in Casualty and General Surgery. | Registrar, Cheltenham General Hospital. |
| Miss M. C. Holderness, M.B., CH.B. | Registrar in Anaesthetics. | S.H.O., Southmead Hospital. |
| A. R. Bollen, M.B., CH.B. | Registrar in Anaesthetics. | Registrar, Royal United Hospital, Bath. |
| D. R. Coles, M.D., CH.B., B.SC. | Hon. Registrar in Medicine and Research Fellow. | |

### *SOUTH-WESTERN REGIONAL HOSPITAL BOARD*

### *BRISTOL*

| *Name* | *Appointment* | *Formerly* |
|---|---|---|
| Cumin, A., M.B., CH.B. (Witwatersrand), M.R.C.P. (London). | Registrar in Chest Diseases, Bristol Chest Clinic and associated hospitals. | Senior Surgeon, P. & O. Company ship. |
| Gall, W. J., M.B., CH.B. (Bristol), F.R.C.S. (England). | Senior Surgical Registrar, Southmead Hospital (joint appt. with United Bristol Hospitals). | Surgical Registrar, Leicester General Hospital. |
| Jancar, J., M.B., B.CH., B.A.O. (N.U.I.), D.P.M. | Assistant Psychiatrist, Stoke Park Hospital Group. | Junior Hospital Medical Officer Stoke Park Hospital. |
| Hancock, D. O., M.B., B.S. (London), F.R.C.S. (Edinburgh). | Registrar in Department of Neurological Surgery, Frenchay Hospital. | House Officer, Accident Service, The Radcliffe Infirmary, Oxford. |

### *SOUTH SOMERSET*

| *Name* | *Appointment* | *Formerly* |
|---|---|---|
| Warboys, J. W., M.D. (Sheffield), D.P.M. | Consultant Psychiatrist, Tone Vale Hospital, near Taunton. | Assistant Psychiatrist, Middlewood Hospital, Sheffield: Physician i/c E.E.G. Dept., Sheffield Royal Infirmary. |
| Collins, W. J. H., M.B., B.CH., B.A.O., D.P.M. | Senior Psychiatric Registrar, Tone Vale Hospital, near Taunton. | Locum S.H.M.O., Warley Hospital, Brentwood. |

### *DEVON AND CORNWALL*

| *Name* | *Appointment* | *Formerly* |
|---|---|---|
| John, H. T., M.B., B.S. (Durham), F.R.C.S. (England) | Senior Surgical Registrar, Royal Devon and Exeter Hospital, Exeter (joint appt. with the United Bristol Hospitals). | Surgical Registrar, University College Hospital. |
| Hackett, A. H., M.B., CH.B. (Otago, N.Z.). | Surgical Registrar, South Devon and East Cornwall Hospital, Plymouth. | S.H.O. in Traumatic Surgery, Birmingham Accident Hospital. |
| McGhie, J. A., M.B., CH.B. (Edinburgh), M.R.C.O.G. | Clinical Assistant in Obstetrics and Gynaecology undertaking eight sessions in the Torquay Group of Hospitals. | Locum Registrar, Royal Devon and Exeter Hospital. |
| Evans, Jill M., M.B., B.CH. (Cambridge), D.R.C.O.G. | Registrar in Obstetrics and Gynaecology, Royal Devon and Exeter Hospital, Exeter. | S.H.O., Pembury Hospital, Kent. |

